# How Long Halt Ye?

**Published:** 1920-12-18

**Authors:** T. Glenton-Kerr

**Affiliations:** The Queen's Hospital for Children, Hackney Road, Bethnal Green, E. 2


### THE EDITOR'S LETTER-BOX.

**Correspondence on all subjects is invited, but we cannot in any way be responsible for the opinions expressed by our correspondents, who must give their name and address as a guarantee of good faith, but not necessarily for publication. Correspondents are reminded that brevity of style and conciseness of statement greatly facilitate early insertion.**

### "How Long Halt Ye?"

*To the Editor of* The Hospital.

Sir,—Mr. Coles states that before making his suggestion that a general voluntary rate on the basis of the National Insurance Scheme should be levied for the hospitals, he carefully reviewed the whole subject, but he has apparently forgotten the existence of the Hospital Saturday Fund, and does not seem to be aware of the fact that the vast majority of the weekly wage earners in London are already contributing generously to hospital funds.

His scheme could only result in existing contributions being diverted into the central fund adumbrated in his letter. It is not to the weekly wage earners that we must look for the required increase of hospital income—for they are already doing their bit—but to the people who are financially above the National Insurance line, and for whom, in the main, no web of collecting machinery exists, as it does in the case of the man of smaller means.—Believe me, yours faithfully,

T. Glenton-Kerr.

The Queen's Hospital for Children,
Hackney Road, Bethnal Green, E. 2,
14th December, 1920.

